# The Pathogenesis of Systemic Sclerosis: The Origin of Fibrosis and Interlink with Vasculopathy and Autoimmunity

**DOI:** 10.3390/ijms241814287

**Published:** 2023-09-19

**Authors:** Junsuk Ko, Maria Noviani, Vasuki Ranjani Chellamuthu, Salvatore Albani, Andrea Hsiu Ling Low

**Affiliations:** 1 Duke-National University of Singapore Medical School, Singapore 169857, Singapore; junsuk_ko@u.duke.nus.edu (J.K.); gmsmari@nus.edu.sg (M.N.); salvo@duke-nus.edu.sg (S.A.); 2 Department of Rheumatology and Immunology, Singapore General Hospital, Singapore 169608, Singapore; 3 Translational Immunology Institute, SingHealth Duke-National University of Singapore Academic Medical Centre, Singapore 169856, Singapore; vasuki.ranjani@visitor.nus.edu.sg

**Keywords:** scleroderma, Raynaud, systemic sclerosis, vasculopathy, inflammation, fibrosis, pathogenesis, autoimmunity

## Abstract

Systemic sclerosis (SSc) is an autoimmune disease associated with increased mortality and poor morbidity, impairing the quality of life in patients. Whilst we know that SSc affects multiple organs via vasculopathy, inflammation, and fibrosis, its exact pathophysiology remains elusive. Microvascular injury and vasculopathy are the initial pathological features of the disease. Clinically, the vasculopathy in SSc is manifested as Raynaud’s phenomenon (reversible vasospasm in reaction to the cold or emotional stress) and digital ulcers due to ischemic injury. There are several reports that medications for vasculopathy, such as bosentan and soluble guanylate cyclase (sGC) modulators, improve not only vasculopathy but also dermal fibrosis, suggesting that vasculopathy is important in SSc. Although vasculopathy is an important initial step of the pathogenesis for SSc, it is still unclear how vasculopathy is related to inflammation and fibrosis. In this review, we focused on the clinical evidence for vasculopathy, the major cellular players for the pathogenesis, including pericytes, adipocytes, endothelial cells (ECs), and myofibroblasts, and their signaling pathway to elucidate the relationship among vasculopathy, inflammation, and fibrosis in SSc.

## 1. Introduction

Systemic sclerosis (SSc) is an autoimmune disease that is characterized by microvascular injury, the dysregulation of adaptive and innate immunity, and the aberrant activation of fibrotic signaling pathways affecting the skin and internal organs [1]. Whilst endothelial dysfunction and widespread microvasculopathy are the hallmark of SSc, large arteries are also increasingly recognized to be part of SSc contributing to coronary artery disease and accelerated atherosclerosis independent of traditional cardiovascular risk factors [2]. Arterial stiffness and damage to elastin fibers were shown to contribute to macrovascular involvement, together with heart valve involvement, thus contributing to increased mortality and poor morbidity and negatively impacting the quality of life in patients [3,4,5,6,7,8,9]. The common cause of death before the advent of established treatments was scleroderma renal crisis (SRC), but pulmonary fibrosis and pulmonary arterial hypertension (PAH) are currently the leading causes of mortality in patients with SSc [7,10,11]. Clinical features of SSc include Raynaud’s phenomenon; skin sclerosis; calcinosis; and gastrointestinal, joint, pulmonary, cardiac, and renal involvement [12]. Clinically, SSc can be classified into diffuse (dcSSc) and limited cutaneous SSc (lcSSc) based on the extent of skin sclerosis [13].

Although SSc classification by cutaneous involvement has discriminatory value in the prognostication of SSc patients, it is limited by the varied, heterogeneous, and overlapping clinical features between the two subsets. In addition, there is a subset called sine scleroderma, in which the patients have no cutaneous involvement but have internal organ manifestations [14]. Furthermore, a subset of patients may also develop overlap syndromes with other connective tissue diseases, e.g., polymyositis and systemic lupus erythematosus [15]. Hence, there is a need to develop a more granular stratification system that could distinctly segregate the different subsets of patients based on pathogenetically homogenous subsets. This would lead to improved prognostication and therapeutic approaches for a better clinical outcome.

In this article, we review the complex pathogenesis of SSc, with particular focus on the origin of fibrosis, as well as its complex interlink with vasculopathy. We also highlight the unmet need for future studies to further untangle the etiopathogenesis of SSc by integrating clinical features with holistic multi-omic approaches. A deeper understanding of etiopathogenesis could lead to the identification of novel therapeutic targets, as well as prognostic and therapeutic clinical biomarkers, towards precision medicine and improved clinical care.

## 2. Vasculopathy as an Initial Step in SSc Pathogenesis

Endothelial injury is an important initial step in the pathogenesis of SSc. Endothelial dysfunction, apoptosis, perivascular inflammation, and platelet aggregation are often found in patients with SSc prior to the onset of disease [16,17,18]. In most SSc patients, Raynaud’s phenomenon (reversible vasospasm of the digits in reaction to the cold or emotional stress) typically appears first before skin sclerosis or involvement of internal organs [19]. The manifestation of Raynaud’s phenomenon and vasculopathy in SSc patients is paralleled by abnormal changes in nailfold capillaries and aberrant immune activation [20,21]. The progressive microvascular damage in the nailfold of patients with Raynaud’s phenomenon predicts the development of definite SSc, suggesting the significant association between early vasculopathy and SSc [19,22]. In addition to the onset of disease, nailfold capillaroscopic pattern and morphology are significantly associated with the severity of both lung and skin fibrosis [23,24,25,26].

## 3. Autoimmunity Link with Vasculopathy

### 3.1. Clinical Evidence

SSc-specific autoantibodies have been described as risk factors for certain organ manifestations, including vascular manifestations. For example, anti-RNA polymerase III antibody is associated with a higher risk of developing gastroesophageal vascular ectasia, PAH, and scleroderma renal crisis [27,28,29,30]. Anti-centromere anti-Th/To, anti-U1 ribonucleoprotein (RNP), and anti-U3 RNP antibodies are associated with a higher risk of PAH [31,32]. In their in vitro studies, Raschi et al. demonstrated the pathogenic role of SSc-specific autoantibodies (anti-Scl70, anti-centromere, and anti-Th/To) embedded in the immune complex in mediating endothelial damage [33], with differential cytokine expression mediated by different autoantibodies. Anti-endothelial cell antibody was previously shown to be associated with digital infarcts and PAH in SSc [34]. The pathogenic role of autoantibody in mediating vasculopathy has been recently demonstrated by Liu et al., as they conducted an in vitro study to investigate the role of autoantibody in connective-tissue-disease-associated PAH [35]. They found that anti-endothelial cell antibodies increased the expression of adhesion molecule ICAM-1 (a marker of endothelial cell (EC) activation) and the production of chemokine RANTES (important chemoattractant for T cells and monocytes) [35]. This study further supports the pathologic function of autoantibody in mediating auto-inflammatory processes, leading to the clinical manifestation of vasculopathy. The autoantibody is likely to be produced by abnormal effector cells; the loss of self-tolerance of B cells could result in the production of autoantibodies [36,37].

The association between the circulating immune cells of patients with SSc and vasculopathic manifestations suggests the role of autoimmunity in SSc [31,34,35,38]. Zhang et al. performed a bioinformatic analysis of gene expression profile data, obtained from the gene expression database to compare the immune signatures across lcSSc patients with PAH and those without PAH, and they observed distinct patterns of immune signatures [38]. A pronounced positive correlation of innate immune cell subsets (monocytes and neutrophils) was noticed in lcSSc patients with PAH, as compared to those without PAH [38]. Furthermore, there was an observed trend of increasing positive correlations between adaptive immune subsets (CD8+ T cells and MAIT (mucosal-associated invariant T) cells) and the innate subsets (dendritic cells and natural killer cells) in lcSSc patients with PAH, as compared to those without PAH [38]. More recently, the role of MAIT cells in SSc and specifically in relation to PAH has been highlighted. MAIT cells, mostly located in mucosal tissues and the liver, are innate-like lymphocytes that have cytotoxic activity and produce proinflammatory cytokines. Peripheral blood MAIT cells were found to be lower in SSc patients than in healthy controls [39,40]. As MAIT cells have the capability of migrating to the site of tissue inflammation, it is possible that the reduced number reflected the recruitment to the site of inflammation [41]. Interestingly, lcSSc patients with PAH had a higher proportion of peripheral blood MAIT cells than healthy controls [38]. These contradictory results could be due to the inhibitory effects of glucocorticosteroid on MAIT cells in other studies and the complex role of MAIT cells in SSc that remains to be elucidated. It is plausible that the circulating immune cell population may play a pathogenic role in the disease pathogenesis underlying vasculopathy. More studies are needed to understand the contribution of innate and adaptive immune subsets in the pathophysiology of SSc, in relation to vasculopathy.

Additionally, medications used for vasculopathy, such as bosentan, clinically improved the modified Rodnan Skin Score (mRSS), although the findings of these studies should be interpreted with caution as the sample size of these open-label studies was small, and the patients were cotreated with other immunosuppressive medication [42,43]. Preclinical in vitro and in vivo studies have demonstrated the direct anti-fibrotic efficacy of soluble guanylate cyclase (sGC) modulators on different fibrotic diseases, including SSc [44]. In a recent phase II clinical trial of riociguat, a sGC stimulator and vasodilator, whilst the primary endpoint of mRSS was not met, the subgroup analysis showed that those with positive anti-RNA polymerase III antibody and negative anti-Scl70 antibody, and with higher baseline mRSS showed significant improvement in mRSS (RISE trial) [45]. The distinct clinical outcome in patients with different autoantibody profiles suggested potentially different pathogenic roles and inflammatory mediators targeted by sGC stimulators. Last but not least, in vitro studies with sildenafil, another medication for vasculopathy, either as monotherapy or in combination with sGC activators, also showed an improvement in the fibrotic phenotype [46,47].

### 3.2. Immunopathogenesis and Interlink with Fibrosis and Vasculopathy

Vasculopathy has been hypothesized to trigger inflammation and fibrosis in SSc. The activation and apoptosis of ECs were found to be mediated by IL-6, suggesting its major role in the early stage of SSc. Indeed, anti-IL-6 receptor antibody tocilizumab improved skin fibrosis in a phase II trial, albeit not statistically significant [48]. Further evidence of the link between immune cell activation and vasculopathy comes from a recent study in which an expansion of angiogenic T cells was observed in SSc patients with severe peripheral vascular complications [49].

Disruption to T-cell homeostasis has been suggested in SSc. Reduced regulatory T cells (Tregs) have been demonstrated in the skin lesions and peripheral blood of patients with SSc [50,51]. Immune polarization in SSc patients was shown to be distinct in different disease stages and statuses [52]. Th2 immune polarization was closely associated with disease exacerbation, while shifts from Th2 to Th1 were observed in parallel with disease duration [52]. In addition, Th17 cells and secreted IL-17 have also been suggested to play an important role in SSc.

The expression of cell adhesion molecules on inflammatory cells and ECs plays a pivotal role in immune polarization. In the peripheral blood mononuclear cells (PBMCs) of SSc patients, the expression of adhesion molecules involved in the tethering to EC and skin homing was elevated [53]. For example, L-selectin, involved in initial tethering to ECs, as well as P-selectin ligand-regulating leukocyte rolling on ECs and T-cell homing to the skin, were elevated, suggesting the ability of SSc inflammatory cells to infiltrate into injured tissues [53]. In the bleomycin-treated mice model, L-selectin and ICAM-1 were shown to regulate Th2 and Th17 cell infiltration, while P-selectin and E-selectin were demonstrated to regulate Th1 cell infiltration [54].

Many studies have found evidence of monocyte/macrophage activation in the fibrotic process, with profibrotic M2 macrophage being the prominent player [55,56,57]. M2 macrophages could secrete IL-13, and the macrophages could also lead to the activation of T cells, which could lead to the production of IL-13, which is profibrotic [58,59]. Another inflammatory mediator of M2 macrophage appears to be IL-6, as M2 macrophage differentiation blockage leads to the reduced secretion of IL-6 [58]. Other cells that produce IL-6 include B effector cells, which have a proinflammatory role [60]. There is a shift in the B-cell homeostasis in favor of more B effector cells and fewer regulatory B cells [60]. Future studies are needed to identify the trigger of this shift. The dysregulation of immune cells is complex, and emerging lines of evidence highlight the roles of plasmacytoid dendritic cells, mast cells, neutrophils, and innate lymphoid cells in immunopathogenesis, as well as fibrosis and vasculopathy [58].

## 4. The Origin of Fibrosis and Interlink with Vasculopathy

### 4.1. Contribution of Pericytes to Fibrosis and Vasculopathy

**Figure 1:**
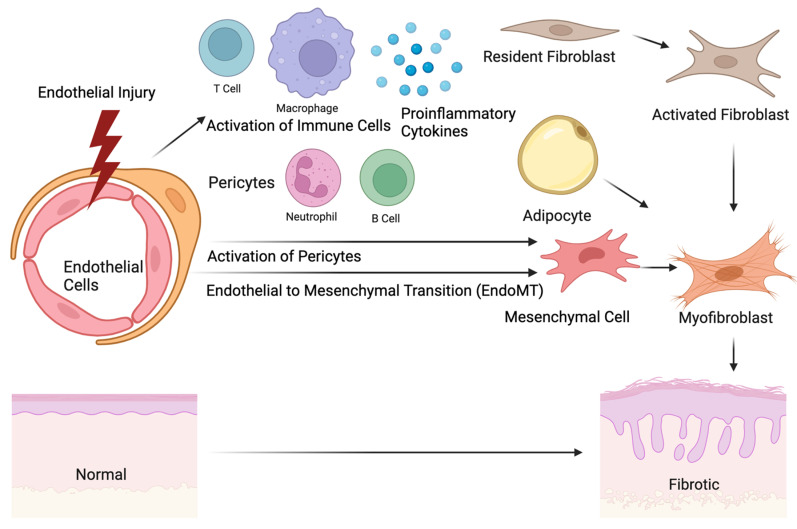
The origin of skin fibrosis in systemic sclerosis. Initial injuries in endothelial cells lead to the recruitment and activation of immune cells, including T cells, B cells, macrophages, and neutrophils. The activation of the immune cells promotes the production of proinflammatory cytokines, which subsequently activate the potential precursors of extracellular matrix (ECM)-producing myofibroblasts, such as pericytes, resident fibroblasts, endothelial cells, or adipocytes. The morphological change in the myofibroblasts and ECM production induce tissue remodeling and skin thickening. The image was created with BioRender.com.

Pericytes are heterogenous perivascular cells mainly residing in the precapillary, capillary, and postcapillary microvasculature (Figure 1) [61]. These cells closely communicate with endothelial, vascular smooth muscle cells, and collagen-producing myofibroblasts for the homeostasis of the vasculature and skin, including vascular permeability, angiogenesis, and blood flow [62]. Although pericytes have been studied extensively in other pathological processes, the current understanding of the role of pericytes in SSc is limited. Pericytes differentiate from myeloid progenitors by transforming growth factor beta (TGF-β), a profibrotic cytokine during development in the skin [63]. In adult skin in vivo, the stimulation of pericytes by TGF-β and platelet-derived growth factor beta (PDGF-β) promotes angiogenesis and the differentiation of pericytes to fibroblasts, suggesting that collagen-producing mesenchymal cells in SSc may be originally from pericytes [64]. This hypothesis is further supported by the overlapping cellular markers of pericytes and myofibroblasts in SSc, such as alpha-smooth muscle cell actin (αSMA), fibronectin (FN), and Thy-1 [65].

How exactly vasculopathy leads to the activation of pericytes is still elusive. Pericytes in immunohistology of skin biopsy samples of patients with early SSc overexpressed PDGF and PDGF receptors, but this was not observed in skin immunohistology from patients with Raynaud’s phenomenon without skin involvement [66]. This result suggests that there might be a phenotypic change in pericytes in relation to vasculopathy in the early pathogenesis of SSc, which later contributes to the development of fibrosis. Indeed, the inhibition of the PDGF signaling pathway attenuates collagen deposition and fibroblast proliferation in the skin, demonstrating that the phenotypic shift in pericytes towards the PDGF signaling pathway is critical for fibrosis [67].

### 4.2. Signaling Pathways Involved in Vasculopathy and Fibrosis

Vasoconstrictors such as endothelin-1 (ET-1) and vasodilators such as nitric oxide (NO) play pivotal roles in vascular dysfunction in SSc patients. ET-1 is elevated in the lungs, kidneys, vasculature, and skin of SSc patients [68,69,70,71]. Conversely, the level of NO is reduced in the vascular endothelium of SSc patients [69,71]. The main source of ET-1 is EC, and ET-1 is a regulator of fibrotic responses, smooth muscle cell proliferation, and vasoconstriction. The level of ET-1 and the clinical severity of skin fibrosis are correlated, suggesting that ET-1 is not only important for vasculopathy but also for skin fibrosis [71,72,73]. One of the key regulators of ET-1 and NO signaling pathways is TGF-β1 [74]. The activation of noncanonical pathways (Smad-independent) not only contributes to the activation of myofibroblasts and ECM production but also the elevation of ET-1 [71,72,75]. This result suggests that fibrosis and vasculopathy are bidirectionally related with ET-1 acting as an amplifying factor.

Multiple signaling pathways have been investigated to elucidate the linkage between the activation of pericytes and vasculopathy. Friend leukemia virus integration 1 (FLI1) is a transcription factor expressed in ECs and is hypothesized to be a negative regulator of skin fibrosis [76]. The expression of Fli1 is decreased in the circulating myeloid in patients with SSc, and the reduction in Fli1 in myeloid cells is associated with profibrotic and proinflammatory phenotypic changes [77]. Furthermore, the decrease in Fli1 is correlated with defective angiogenesis, the loss of pericytes in the vasculature, fibrosis, and immune system abnormalities [78,79]. The conditional deletion of Fli1 in EC recapitulates, at least in part, the phenotypes of SSc, including vasculopathy, impaired angiogenesis, and the activation of fibroblasts in mice [76,80]. Epigenetic modifications, such as DNA methylation and histone acetylation, and miRNAs are hypothesized to be upstream of the *FLI1* gene reduction in SSc [81,82]. Despite the remarkable findings from in vivo animal models of Fli1 deletion and skin fibrosis, the expression pattern of Fli1 in patients with SSc has not been well characterized, and it is unclear which cell types show a reduction in Fli1 expression leading to the development of fibrosis in patients with SSc.

In addition to Fli1, caveolin-1, a membrane protein critical for the formation of vesicles and membrane invagination, has been proposed to be an important player in SSc. The caveolin-1 rs959173C minor allele is correlated with a reduced risk of SSc in Caucasian populations, suggesting that the polymorphism of caveolin-1 can alter the susceptibility to SSc [83]. Caveolin-1 is markedly reduced in both skin and lung biopsy samples isolated from patients with SSc [84]. Additionally, bone marrow mesenchymal cells in SSc patients show a significant reduction in caveolin-1 and are associated with the profibrotic phenotype [85]. Caveolin-1-dependent invaginations reduce TGF-β1 signaling significantly as a result of the internalization of the TGF-β1 receptor [84]. The deletion of caveolin-1 in mice is sufficient to induce the impairment of vascular tone, and spontaneous endothelial-to-mesenchymal transition, and to promote lung and skin fibrosis [84,86]. Deficiency in caveolin-1 in mesenchymal cells seems to upregulate vascular endothelial growth factor A (VEGF) signaling, which is implicated in SSc [87]. In summary, caveolin-1 is one of the important mediators between vasculopathy and fibrosis. However, the exact connection of the differential expression of caveolin-1 in ECs and pericytes with vasculopathy and fibrosis needs to be further investigated.

### 4.3. Role of Adipocytes in Fibrosis and Vasculopathy

Adipocytes are the cells primarily composing adipose tissues and serve as an important reservoir of energy and fat. There is increasing evidence that adipocytes may play a role in the pathogenesis of skin fibrosis. The loss of subcutaneous adipocytes is one of the hallmarks of SSc [88]. This phenomenon is consistently found in various animal models for skin fibrosis, including bleomycin-induced [89], angiotensin II-induced [90], TGF-β-induced [91], and tight skin models [92].

A tdTomato-based lineage study on adiponectin, a marker of adipocytes, demonstrated that adiponectin positive cells differentiated into myofibroblast-like cells upon fibrotic injuries. Moreover, the unique gene profile of adipocytes decreased before the induction of profibrotic gene transcripts, suggesting that adipocytes might be the origin of collagen-producing fibroblasts in skin fibrosis [93,94]. A reduction in proliferator-activated receptor γ (PPAR-γ), a well-known nuclear hormone receptor for adipogenesis, and activation found in inflammatory zone 1, FIZZ1, have been proposed as a trigger of the trans-differentiation of adipocytes to myofibroblasts [95,96]. However, the exact mechanism of phenotypic changes in adipocytes in SSc is still elusive.

The interplay between adipocytes and vasculopathy is an active area of research. Adipocytes produce a variety of adipokines activating various cell types via autocrine, paracrine, and endocrine pathways. An altered balance of adipokine due to adipocyte loss or transition to fibroblasts may contribute to inflammation, vasculopathy, and fibrosis [97,98,99,100,101]. Adiponectin, one of the SSc-related adipokines, has a protective role in the pathogenesis of SSc; mice with deleted adiponectin develop less fibrosis upon bleomycin challenge [102]. Furthermore, in a bleomycin model of skin fibrosis, a pharmacological intervention not only reduced the degree of fibrosis but also inflammation via the inhibition of the transition from adipocytes to myofibroblasts, suggesting that adipokines may be one of the interlinks between inflammation and fibrosis [103].

Interestingly, fat graft induces dermal adipose regeneration and reduces skin fibrosis in both SSc patients [104] and animal models of skin fibrosis [105]. Furthermore, the grafting of autologous adipose tissues improves the treatment-resistant digital ulcers, demonstrating that vasculopathy and ulcerations are presumably downstream of the adipose pathology in SSc [106]. Although the therapeutic effect of adipocyte graft and how exactly adipose tissues contribute to the development of vasculopathy and fibrosis should be further investigated, this evidence significantly suggests that adipocytes play a key role in the pathogenesis of SSc.

### 4.4. Fibrocytes

Bone marrow-derived fibrocytes have been investigated as a potential origin in SSc. These cells are fibroblast-like cells expressing collagen 1 and increase in number in the event of acute injury and fibrosis [107]. Patients with interstitial lung disease (ILD) secondary to SSc have an increased number of circulating fibrocytes [108]. Furthermore, the severity of the fibrosis is correlated with the number of circulating fibrocytes in lcSSc [109]. Although there are a limited number of studies available for SSc regarding fibrocytes and bone marrow-derived collagen-positive cells, a bone marrow transplant study demonstrated that very few collagen-positive cells were positive for platelet-derived growth factor receptor alpha (PDGFR-α), a marker of fibroblasts derived from bone marrow [110]. This suggested that the collagen-positive cells were very likely originally from local cells residing in the skin.

### 4.5. Endothelial-to-Mesenchymal Transition (EndoMT)

**Figure 2:**
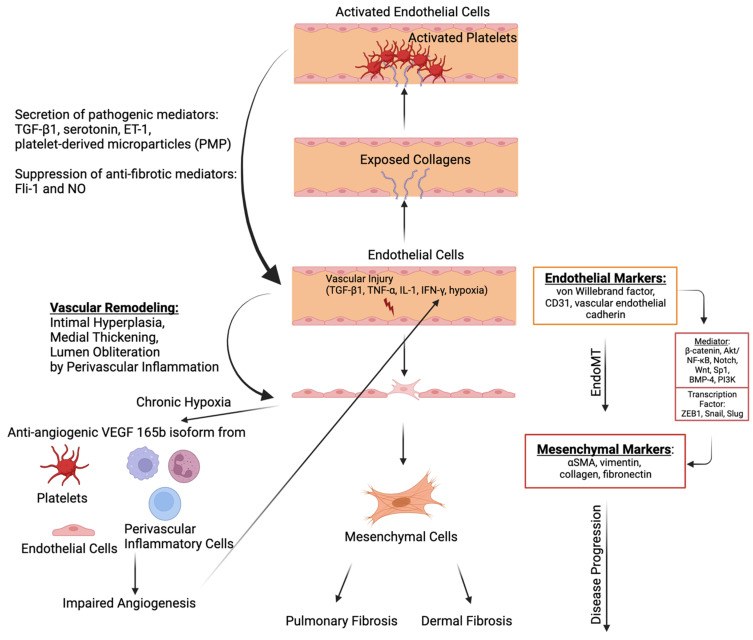
The molecular mechanism of endothelial-to-mesenchymal transition (EndoMT) and its interlink with fibrosis. Factors that contribute to vascular injuries like transforming growth factor beta 1 (TGF-β1), tumor necrosis factor alpha (TNF-α), IL-1, interferon-gamma (IFN-γ), and hypoxia induce the transition to mesenchymal cells via various mediators (e.g., β-catenin, Akt/NF-kB, Notch, Wnt, Sp1, BMP-4, and PI3K) and subsequent elevation of transcription factors (ZEB1, Snail, and Slug). Furthermore, vascular injuries promote vascular remodeling, including intimal hyperplasia, medial thickening, and lumen obliteration, through perivascular inflammation. Along with this vascular remodeling, chronic hypoxia increases anti-angiogenic vascular endothelial growth factor 165b (VEGF165b) isoform from platelets, endothelial cells, and immune cells, including macrophages, leading to perpetual hypoxia. Mesenchymal cells contribute to pulmonary and dermal fibrosis by producing extracellular matrix (ECM), including fibronectin and collagen. On the other hand, vascular injuries can lead to the activation of platelets via the exposure of collagens in the endothelium. The activated platelets and endothelial cells secrete profibrotic mediators, inducing TGF-β1, serotonin, endothelin-1 (ET-1), and platelet-derived microparticles (PMPs), which further amplify the fibrosis in SSc. Furthermore, anti-fibrotic mediators, including Fli-1 and nitric oxide (NO), are suppressed. The image was created with BioRender.com.

EndoMT is a phenomenon of cellular trans-differentiation by which ECs lose vascular EC markers (e.g., von Willebrand factor, CD31, and vascular endothelial cadherin) and gain mesenchymal cell markers (e.g., αSMA, vimentin, collagen, and FN; Figure 2) [111,112]. EndoMT is mediated through various signaling molecules, including β-catenin, Akt, nuclear factor kappa-light-chain-enhancer of activated B cells (NF-kB), Notch, Wnt, Sp1, bone morphogenetic protein 4 (BMP-4), and phosphoinositide 3-kinase (PI3K) [113]. These mediators lead to the elevation of transcription factors, including ZEB1, Snail, and Slug, which subsequently induce the expression of target genes related to mesenchymal cells [113]. ECs lose the typical cobblestone morphology and acquire the phenotypic profile and proliferative ability of mesenchymal cells. During EndoMT, EC disaggregation from the vessel lining leads to an impaired vessel layer [114]. This EndoMT process is observed during wound healing but also during pathological processes of vascular injury with characteristic fibrosis and inflammation [111].

In SSc vasculopathy, the small and medium-sized arteries may undergo intimal hyperplasia, leading to medial thickening and lumen obliteration, contributed by perivascular inflammation and microthrombi [115]. Although microvasculature loss was observed during SSc with persistent hypoxia, compensatory angiogenesis did not occur [116]. It was previously demonstrated that although hypoxia promotes the release of vascular endothelial growth factor (VEGF), impaired angiogenesis persists, and this could be due to anti-angiogenic VEGF 165b isoform overexpressed in ECs, fibroblasts, and inflammatory cells in SSc [117]. Furthermore, platelets might also release VEGF165b after activation with the damaged endothelia [118].

The pivotal inducer of EndoMT is postulated to be the TGF-β family [69]. TGF-β promotes morphological changes in ECs, leading to the reduced expression of ECs and increased expression of mesenchymal markers [119,120]. The secretion of TGF-β and its synthesis may be increased by endothelin-1 (ET-1), and ET-1 has been shown to have a synergistic effect with TGF-β to modulate EndoMT [121,122]. TGF-β activates the canonical Wnt pathway, and Wnt3a may play a role in EndoMT by promoting the expression of cadherin and inducing the expression of vimentin [123]. Other possible mediators of EndoMT include tumor necrosis factor alpha (TNF-α), which enhances TGF-β-induced EndoMT by stimulating the TGF-β signaling pathway [124]. Moreover, interferon-gamma (IFN-γ) has also been reported to mediate EndoMT via TGF-β2 and ET-1 signaling pathways in SSc [125].

EndoMT could potentially be a therapeutic target for fibrotic disease as evidenced by preclinical studies utilizing anti-vasculopathy treatment. ET-1 receptor antagonist macitentan has been reported to inhibit both ET-1-induced and TGF-β-induced EndoMT in microvascular ECs isolated from SSc patients [121,126]. These findings were confirmed by other in vitro studies using bosentan, another ET-1 receptor antagonist, in the fibroblast and EC co-culture model [127]. Iloprost, which is an analogue of prostacyclin, promotes VE-cadherin clustering and stability at the adherent junction, promotes angiogenesis, and prevents EndoMT [128]. A better understanding of the pathophysiology underpinning EndoMT has opened up potential therapeutic avenues for the development of anti-fibrotic therapies. More studies are needed to investigate the efficacy of EndoMT inhibitors in SSc.

### 4.6. Platelet Activation in the Interlink between Vasculopathy, Autoimmunity, and Fibrosis

Endothelial injury through the exposure of collagen from the subendothelial matrix contributes to the activation of platelets, which have been found to be actively involved in the pathogenesis of SSc [129,130,131]. Platelet activation led to the release of profibrotic mediators such as TGF-β and serotonin. Platelet-derived serotonin was shown to strongly induce extracellular matrix synthesis in a TGF-β-dependent manner. The inactivation of serotonin was demonstrated to prevent the onset of fibrosis and ameliorate established fibrosis. In addition to being a source of profibrotic signals, activated platelets produced microparticles [132]. Platelet-derived microparticles (PMPs) were shown to be associated with clinical features in SSc. PMP levels were significantly higher in patients with disease duration >3 years and in patients with positive anti-topoisomerase-I antibodies [133]. Prior studies demonstrated that PMPs promoted neutrophil autophagy, induced neutrophil activation, and enhanced neutrophil extracellular trap (NET) production [134]. In a study by Didier et al., it was shown that NET production by polymorphonuclear neutrophils (PMNs) from SSc patients with severe vascular complications (PAH, digital ulcer) was higher than those without severe vascular complications [135]. Additionally, platelets modulate immune responses by interacting with Tregs and activating monocytes or B cells via the costimulatory axis CD40/CD40L [129]. The growing insights into the potential contribution of platelets in the vicious cycle of fibrosis, autoimmunity, and vascular damage SSc highlight potential novel therapeutic interventions.

## 5. Unmet Needs and Future Studies

At the molecular level, SSc is a heterogeneous disease with varying clinical outcomes. The crude clinical classification into dcSSc and lcSSc is insufficient to reflect this heterogeneity. Whilst acknowledging that different SSc-specific autoantibodies are associated with distinct clinical phenotypes and organ involvement in SSc, there remains a great unmet need for precision medicine to aid targeted treatment based on patients’ dynamic biological state. Layered upon the possibility of distinct subtypes of SSc with distinct clinical outcomes, patients may transition through different stages of disease such as a predominant vasculopathic or inflammatory stage to the fibrotic stage. For example, in a phase II trial of abatacept, a T-cell inhibitor, whose primary endpoint of skin fibrosis improvement was not met, patients’ skin biopsies with the inflammatory gene subset showed significant improvement in mRSS compared with those of the fibro-proliferative subset [136]. In contrast, patients with fibro-proliferative gene signatures, but not the inflammatory gene signatures, were found to respond to tyrosine kinase inhibitor nilotinib, imatinib, and dasatinib [137,138,139]. These serve as proof of concept to utilise molecular phenotyping to guide treatment approaches by selecting treatment for patients who are most likely to respond towards precision medicine.

The advent of multi-omic platforms has opened possibilities to gain deeper insights into the molecular mechanisms underlying SSc. Future direction necessitates a holistic approach to integrate these data from various omic platforms (transcriptomics, genomics, proteomics, cytomics, epigenomics, and microbiomics) to shed light on the signalling pathways underlying the complex etiopathogenesis [13]. For example, although the role of activated macrophages has been implicated in the regulation of inflammation, fibrosis, and vascularisation, the trigger that underlies aberrant macrophage activation is not clear [140]. The integration of clinical phenotyping with a multi-omic approach suggested the important role of IL-13 in monocyte–macrophage activation in the development of PAH in SSc [141]. Christmann et al. investigated the monocyte–macrophage activation in patients with SSc-PAH by combining transcriptomics, proteomics, and cytomics; the results revealed the upregulation of MRC1 (c-type mannose receptor 1, a marker of alternative activation of monocyte–macrophage) expression in CD14+ cells, and this was greatly increased upon stimulation with IL-13, the concentration of which was most increased in patients with lcSSc-PAH [141,142]. More recent investigations have highlighted the important role of epigenetic factors in regulating gene expression in SSc, specifically through DNA methylation, hydroxymethylation, histone modification, and noncoding RNAs without modifying the underlying DNA sequences [143,144]. Epigenetic modification by environmental signals is implicated in the pathogenesis of SSc in genetically susceptible individuals [144]. Advances in technologies, such as cytometry by time of flight (CyTOF), are promising with the possibility to now look at cellular markers in whole blood, including platelet and red blood cells [145,146,147]. Furthermore, the Extended Polydimensional Immunome Characterization (EPIC), a web-based tool, could be used for the analysis of high-dimensional biomarkers in SSc patients compared with datasets of healthy controls [148]. The discovery of biomarkers for early diagnosis, patient stratification, monitoring disease progression, and treatment is sorely needed.

In this paper, we highlighted the origin of fibrosis, and the complex interplay between inflammation, vasculopathy, and fibrosis. Research in SSc is challenging due to its heterogeneous nature and rarity. Limitations in sample size and lack of complete clinical information in published data hindered data analysis and interpretation to a great extent. Overcoming these limitations requires a collaborative effort among clinicians and scientists. Furthermore, a holistic approach to address research questions by deploying the current technological advancement to integrate clinical features with multi-omic advances is crucial. Such a strategy holds promise for improving SSc management and tailored treatment for individual patients.
